# Editorial: Climate Change and Plant Nutrient Relations

**DOI:** 10.3389/fpls.2020.00869

**Published:** 2020-06-30

**Authors:** Scott Heckathorn, Gretchen North, Dan Wang, Chunwu Zhu

**Affiliations:** ^1^Department of Environmental Sciences, University of Toledo, Toledo, OH, United States; ^2^Department of Biology, Occidental College, Los Angeles, CA, United States; ^3^International Center of Ecology, Meteorology and Environment, College of Applied Meteorology, Nanjing University of Information Science and Technology, Nanjing, China; ^4^State Key Laboratory of Soil and Sustainable Agriculture, Institute of Soil Science, Chinese Academy of Sciences, Nanjing, China

**Keywords:** climate change, nutrients, nutrition, plants, food quality

Plants are experiencing significant environmental change due to human activities, including climate change driven by increases in the concentration of atmospheric carbon-dioxide and other greenhouse gases, which is increasing both average and extreme high temperatures and altering precipitation patterns. Against the backdrop of climate change, humans are also impacting global nutrient cycles, such as by increasing the amount of available nitrogen (N) via increases in the use of N fertilizers and the farming of leguminous crops. These human-caused environmental changes will likely grow larger, and their rate of change will likely accelerate, in the coming decades. Understanding the mechanisms by which plants respond and adapt to these new environmental challenges is crucial, in order to develop adaptive strategies for maintaining agricultural productivity and ecosystem services. Importantly, it is clear that nutrient availability affects plant responses to climate change, but our knowledge in this area is insufficient. To raise awareness of, and stimulate research in, this field, this Research Topic focused on how plant nutrition interacts with climate change to affect plant function. The eight articles published in this Research Topic investigated different aspects of climate change and nutrition that ranged from interactive effects of N and elevated CO_2_ (eCO_2_) or N and water stress on various aspects of plant form and function, to effects of eCO_2_ on the quality of plant tissue as food or on nutrient re-translocation within plants.

The articles by Nielsen et al. and Ayi et al. deal with two contrasting environmental challenges that are worsening due to climate change: drought and flooding, respectively, conditions that are almost always accompanied by nutrient stress. In their study of the interactive effects of water and nutrient availability, Nielsen et al. take as their subjects three congeneric pairs of species native to semi-arid habitats in California, one member of each pair from serpentine soil (typically high in Mg and low in Ca, N, *etc*.) and thus assumed to be tolerant of low soil nutrients paired with one from non-serpentine soil. The authors hypothesized that the non-serpentine species will be tolerant of a broader combination of nutrients and water largely because of their faster growth and ability to adjust more quickly than the serpentine species. In addition, they hypothesized that all species would fare better in high water availability and nutrients than in low. In a greenhouse study, they measured plant growth responses in the context of functional traits (e.g., relative growth rate, root mass ratio, photosynthetic nitrogen-use efficiency), and one of their salient results is that functional traits based on nutrient use and allocation explained more of the response variability than did other traits. Moreover, contrary to hypothesis, they found that no matter their origin, species responded most favorably to a combination of low water and high nutrients. One conclusion is that under conditions of increased rainfall, native plants may actually grow more poorly. In keeping with their hypothesis, the non-serpentine species exhibited greater plasticity, suggesting that climate variability will favor plants that grow relatively quickly. In contrast, Ayi et al. investigated a widespread and often invasive plant, *Alternanthera philoxeroides*, or alligator-weed, which is invasive along waterways and is tolerant of flooding. In separate hydroponic experiments, they measure plant growth and root anatomical change in response to varying concentrations of oxygen or nutrients. The authors focused on root efficiency, which they defined as total plant biomass divided by root surface area. As hypothesized, root efficiency generally decreased in response to decreasing nutrients, as plants allocated more biomass to roots, chiefly by forming longer thinner roots, leading to increased root surface area. Plant responses to reduced oxygen concentration were less expected; specifically, root efficiency was highest under the lowest concentration of oxygen. The authors attribute this result to the larger-diameter air channels that developed in roots under low oxygen, a finding that may be relevant to flooding-tolerant crops such as rice.

The Xu et al. and Hasegawa et al. articles investigated the interactive effects of variation in carbon and nitrogen availability, which is essential to understanding how plants will grow in an eCO_2_ world and which have significant impacts on plant-animal interactions. The Xu et al. paper found that one rice cultivar, Takanari, retained its high-yielding advantage over other cultivars examined at eCO_2_, even under limited N conditions. The paper suggested that this cultivar could be a useful genetic resource for improving N-use efficiency under eCO_2_ in the future. The Hasegawa et al. paper tested the combined effects of eCO_2_ and increased N fertilization on interactions between maize and an insect herbivore, *Ostrinia furnacalis*. They found that eCO_2_ and increased N fertilization boosted carbon and nitrogen metabolism and promoted levels of plant defensive compounds in maize (with or without *O. furnacalis*-induced production), which ultimately decreased the fitness of *O. furnacalis* to the host. Despite the significant findings with these two papers, more studies including multiple crop species and cultivars are highly needed to understand the metabolic responses of plants to eCO_2_ and increased N fertilization, and how this impacts plant-herbivore interactions in the context of future climate-change scenarios.

The articles by Li Y. et al. and Dong et al. added important information to our understanding of how the growth of plants in eCO_2_ affects their nutritional quality as food. Declines in the concentration of protein and minerals essential for humans, including iron and zinc, have been reported for key food crops when grown in eCO_2_. For the current century, initial estimates of the potential human health impact of such declines in plant food quality exceed more than 100 million persons affected, depending on the nutrient in question. However, until now, a comprehensive assessment of the potential changes in the food quality of vegetables in response to eCO_2_ has been lacking. The article by Li Y. et al. examines effects of eCO_2_ on nutrient content of soybean seeds (which is often eaten as a vegetable), while the article by Dong et al. describes results of a meta-analysis to quantify the effects of eCO_2_ on nutrient content of other vegetables. Overall, their results indicate that the role of rising eCO_2_ in altering vegetable food quality may represent an under-appreciated human health effect associated with anthropogenic climate change. They also noted that there are widely varied responses of nutrients to eCO_2_ among species and varieties of vegetables. These results high-light the urgent task that plant scientists face, as far as characterizing the effects of climate change on plant food quality and then helping to develop efficient ways to mitigate the decline of key nutrients in plant tissue due to eCO_2_ (e.g., with crop breeding or genetic engineering, fertilizer applications, or changes in cultivation techniques).

Finally, Crous et al. and Li L. et al. examined effects of eCO_2_ on N (or N and phosphorus, P) resorption from leaves in trees. Using FACE technology, Crous et al. grew Eucalyptus species in eCO_2_ for 5 years, and then examined eCO_2_ effects on %N and %P of plant tissues, as well as the resorption of N and P from leaves to stems. In this forest, which is P-limited more than N-limited, eCO_2_ had little effect on leaf %P, and it decreased leaf %N significantly only in young leaves. Further, at senescence, retranslocation of P and N from leaves to wood was not affected by eCO_2_. Trees in this nutrient-limited forest were flexible as far as their N and P stoichiometry, which enabled them to minimize common effects of eCO_2_ on leaf nutrient relations. Meanwhile, Li L. et al. examined effects of eCO_2_ on leaf N resorption during autumnal senescence in *Tilia americana*, using eCO_2_ treatments in greenhouses. They found that eCO_2_ hastened the formation of overwintering buds and the start of leaf senescence, while extending the time that it took leaves to senescence. eCO_2_ also increased N-resorption efficiency from leaves. Hence, climate change will have a dramatic impact on autumnal senescence in this species in the field, as it is expected to have for other tree species.

In closing, these articles in this Research Topic add to our knowledge in the area of climate change and plant nutrient relations, while illustrating the need for more such research, such as the need for studies that examine interactive effects of multiple climate-change factors, impacts on wild species and woody species, and effects on interactions between plants and their partners or enemies.

## Author Contributions

All authors listed have made a substantial, direct and intellectual contribution to the work, and approved it for publication.

## Conflict of Interest

The authors declare that the research was conducted in the absence of any commercial or financial relationships that could be construed as a potential conflict of interest.

